# Winchester Nurses' Home

**Published:** 1911-09-09

**Authors:** 

**Affiliations:** Architects and Surveyors. 24 Portland Street, Southampton.; Architects and Surveyors. 24 Portland Street, Southampton.


### INSTITUTIONAL LETTER-BOX.

### WINCHESTER NURSES' HOME.

*To the Editor of* The Hospital.

Sir,—We should like to say that as a result of your article (The Hospital, August 26, page 552) we have had inquiries from the secretary of a well-known hospital near London as to the cost and other particulars, and we should be glad if you will publish our reply to some of the criticisms you have made.

First, the ventilation and heating. In each room not provided with a fireplace there is a radiator, Tobin-tube inlet ventilators are built into the external walls, and over each bedroom door a fanlight opening into the main corridor, so that good through ventilation is provided.

The sisters' bed-sitting-room is divided into two equal parts by a curtain. It is a matter of opinion whether a second door would not have been an improvement, but we know the arrangement, as planned, works very well in practice.

There is a slop sink on both floors fitted in the room called housemaids' pantry. The building provides accommodation for twenty nurses and two sisters, and cost £3,266—£148 per nurse. We may also say we obtained the job in public competition against five other selected architects.—Yours faithfully,

Weston and Burnett.

24 Portland Street, Southampton. Architects and Surveyors.

